# Alcyonacea: A Potential Source for Production of Nitrogen-Containing Metabolites

**DOI:** 10.3390/molecules24020286

**Published:** 2019-01-14

**Authors:** Walied Mohamed Alarif, Ahmed Abdel-Lateff, Hajer Saeed Alorfi, Najla Ali Alburae

**Affiliations:** 1 Department of Marine Chemistry, Faculty of Marine Sciences, King Abdulaziz University, PO. Box 80207, Jeddah 21589, Saudi Arabia; 2 Department of Natural Products and Alternative Medicine, Faculty of Pharmacy, King Abdulaziz University, PO Box 80260, Jeddah 21589, Saudi Arabia; 3 Department of Pharmacognosy, Faculty of Pharmacy, Minia University, Minia 61519, Egypt; 4 Department of Chemistry, Faculty of Science, King Abdulaziz University, PO. Box 80203, Jeddah 21589, Saudi Arabia; halorfi@kau.edu.sa; 5 Department of Biology, Faculty of Science, King Abdulaziz University, PO. Box 80203, Jeddah 21589, Saudi Arabia; nalbourai@kau.edu.sa; 6 Biology Department, College of Science, Princess Nourah bint Abdulrahman University, PO. Box 84428, Riyadh 11671, Saudi Arabia

**Keywords:** Alcyonacea, sesquiterpenes, diterpenes, antitumor, cytotoxicity

## Abstract

Alcyonacea (soft corals and gorgonia) are well known for their production of a wide array of unprecedented architecture of bioactive metabolites. This diversity of compounds reported from Alcyonacea confirms its productivity as a source of drug leads and, consequently, indicates requirement of further chemo-biological investigation. This review can be considered a roadmap to investigate the Alcyonacea, particularly those produce nitrogen-containing metabolites. It covers the era from the beginning of marine nitrogen-containing terpenoids isolation from Alcyonacea up to December 2018. One hundred twenty-one compounds with nitrogenous moiety are published from fifteen genera. Their prominent biological activity is evident in their antiproliferative effect, which makes them interesting as potential leads for antitumor agents. For instance, eleutherobin and sarcodictyins are in preclinical or clinical stages.

## 1. Introduction

The marine biota is characterized by living under harsh environmental conditions (e.g., high salinity, variable pressures, hydrothermal vents and variable nutrient accessibility) [1,2]. The marine invertebrates, particularly soft corals, suffer from absence of mechanical defenses [3,4,5]. They produce chemical agents to maintain their life. These defenses can be represented by production of secondary metabolites that possess ecological functions including anti-predatory protection, thus several natural metabolites were discovered from marine sources [6]. Most of these substances have unprecedented structures with diversity of pharmacological application [7,8,9,10,11,12,13]. Alcyonacea comprises marine invertebrates that live ubiquitously in tropical sea waters, particularly intertidal zones or inner reefs below the stony corals, and are less prone to damage from collecting or shipping than the stony corals [14]. They are animals, provide stinging cells in the form of toxic stinging nematocysts with absence of the rigid protective skeleton of scleractinians, and possess allelopathic capabilities of chemical production [15,16]. This enables the sessile corals to strive and reduce their palatability, by reducing fouling effect through producing chemical substances, mucus or terpenoids, aiming at protecting them against predators [17,18,19]. Alcyonacea is well known for the production of terpenoids, while the occurrence of nitrogenous terpenoidal derivatives is rare. The bio-synthetic pathway of the nitrogenous moiety is unclear [6,20,21]. Conclusively, Alcyonacea has conceivable therapeutics, which includes immunomodulator, anticancer and useful antifouling agents. 

Amazingly, 40% of the chemical frameworks that appear in different databases are natural compounds. Approximately, half of new drugs recently reported are of natural origin or constructed based on natural architectures [22]. A comparative analysis study indicates that marine products are superior to earthly metabolites in terms of chemical uniqueness [23]. An investigation has led to establishing that 70% of metabolites that appear in Dictionary of Marine Natural Products (DMNP) are completely exploited by marine invertebrates. Additionally, marine drugs have successfully launched in the market, and others are still in different phases of clinical trials. A recent review reports the marine pharmaceutical products [24,25,26]. 

In 1969, FDA approved cytarabine as an anticancer agent, while vidarabine was permitted in 1976 as an antiviral agent. Around fourteen years later, ziconotide was approval by FDA for treatment of severe chronic pain. Trabectedin was approved from FDA in 2010 for metastatic breast cancer [25]. On their way to the market, another twelve marine-derived drugs are being clinically investigated [26]; for instance, the bryostatin and dolastatin derivatives, soblidotin and synthadotin, respectively. A tricyclic diterpenoid, eleutherobin, has been reported from *Eleutherobia* sp., with potent inducer of tubulin polymerization in vitro, which mimics taxol-like effect [25,26,27,28]. 

In the current review, marine nitrogen-containing metabolites isolated from Alcyonacea are presented. These compounds show significant effects toward certain diseases and/or have a role in drug discovery. It is interesting to discuss the future perspectives of the structure–activity relationship of these metabolites. An extensive bibliographic literature survey was conducted employing different scientific databases including Scopus, Pubmed, SciFinder, Google scholar and Web of Science.

## 2. Chemical Constituents

### 2.1. N-Containing Sesquiterpenes

**Figure 1:**
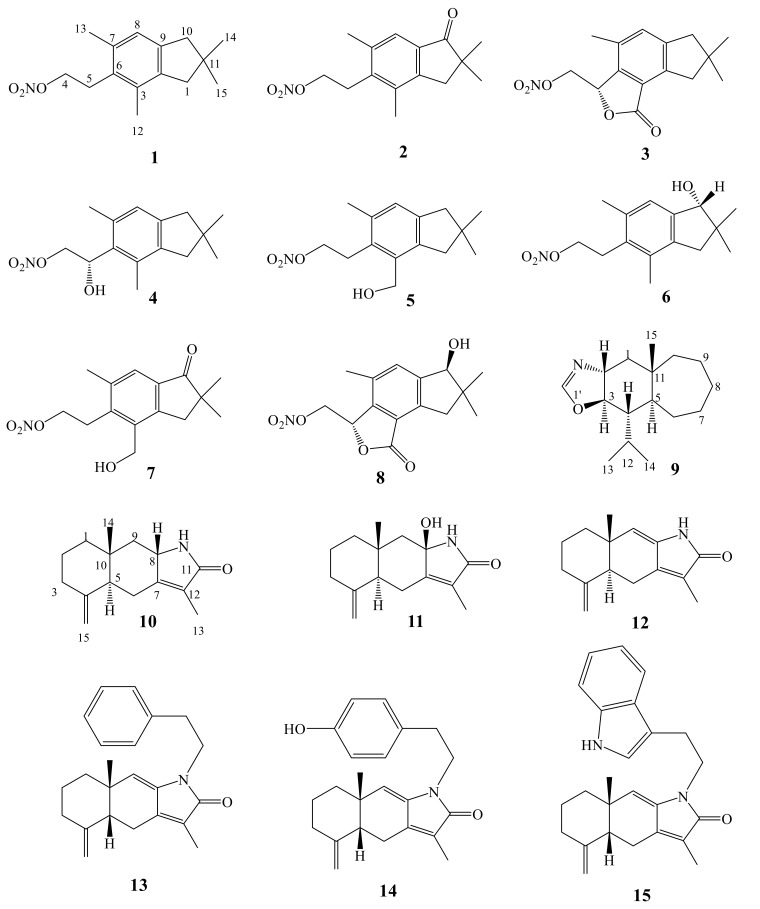
N-Containing sesquiterpenes (**1**–**15**).

Sesquiterpenes are a class of widely distributed metabolites among marine soft corals. Although nitrogen-containing sesquiterpenoids are rare, they are produced by certain soft corals (e.g., *Alcyonium* sp.) [20]. Illudalane-type sesquiterpenoids (Table 1 and Figure 1), have limited distribution in nature and metabolites of this class are exclusively reported from ferns and fungi [29,30]. Congeners of illudalane-type sesquiterpenoids identified as alcyopterosins (B, C, E, F, G, H, J and M) (**1**–**8**) are reported from the sub-Antarctic globular spongy pink coral *Alcyonium paessleri*, gathered around South Georgia Islands. The combined extracts (EtOH and EtOAc) purified by employing different chromatographic techniques led to isolation of the congeners (**1**–**8**), which numerated as the first report of nitrogen-containing illudalane type sesquiterpenoids, as well as a first report of nitrate ester to be isolated from marine soft corals [20]. These metabolites represent a new trend in biosynthetic pathway of sesquiterpenoids. All reported sesquiterpenes may have sulfate, phosphate, or halogenic moieties, but finding nitrogen-containing sesquiterpenes is infrequent. Alcyopterosins (**1**–**8**) were evaluated for their cytotoxicity towards Human larynx carcinoma (Hep-2) and Human colon carcinoma (HT-29). Alcyopterosins E (**5**) showed cytotoxicity against Hep-2 with IC_50_ value of 13.5 µM, while Alcyopterosins C (**2**) and H (**6**) showed cytotoxicity against HT-29 with IC_50_ value of 10 µM [20]. Although nitrate ester derivatives are very rare, some synthetic nitrate esters are published. An acceptable speculation is that the hydrolysis of esters leads to the corresponding alcohols, which play roles in their cytotoxic effects [20].

Cladioxazole (**9**) represents the first oxazole-derived terpenoid, isolated from *Cladiella* sp. which, collected from Andaman Island [21]. Unfortunately, no information about the biological activity of **9** has been published. Three sesquiterpene lactams, taenialactams A (**10**), B (**11**) and atractylenolactam (**12**), were isolated from *Cespitularia taeniata* collected from Taiwan [31]. The methanolic extract of *C. taeniata* has been partitioned between water and ethyl acetate. The EtOAc-soluble portion has been purified on silica gel columns and HPLC to yield **10**–**12**. Atractylenolactam (**12**) was previously isolated from the rhizome of a terrestrial plant, *Atractylodes macrocephala*, a Chinese medicinal plant employed for the treatment of indigestion and anorexia [32]. Compounds **10**–**12** were evaluated towards KB, WiDr, and Daoy tumor cells, no effects were observed [31]. Fractionation of the organic extract of Taiwanese soft coral *Cespitularia taeniata*, by employing different chromatographic techniques including HPLC led to identification of ten compounds, three of which are nitrogenous sesquiterpenoids, cespilamides C–E (**13**–1**5**, Figure 1). Cespilamide E (**15**) exhibited cytotoxicity against Hela, Daoy, WiDr and MCF-7 cells with IC_50_ of 24.7, 22.3, 34.1 and 17.5 μM, respectively. Cespilamide E (**13**) exhibited cytotoxicity against Hela, Daoy, WiDr and MCF-7 cells with IC_50_ of 30.9, 34.8, 49.5 and 30.6 μM, respectively, while cespilamide D (**14**) was inactive (>40 μM) [31,33].

### 2.2. N-Containing Diterpenes

**Figure 2:**
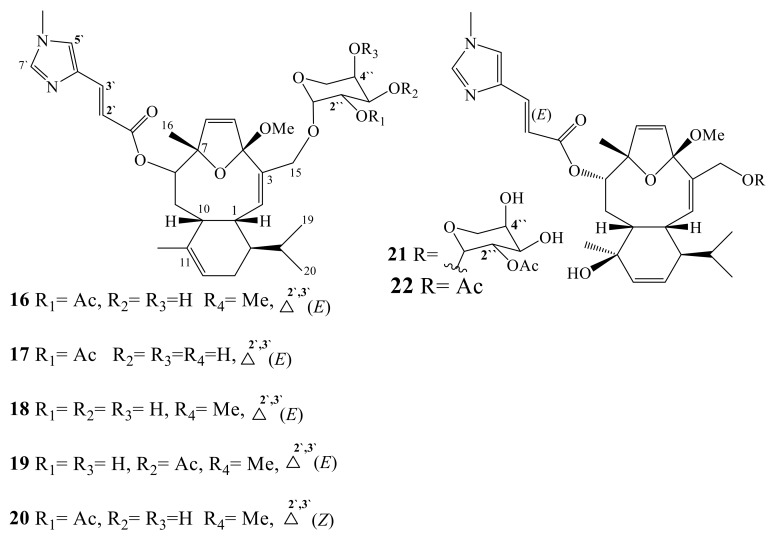
N-Containing diterpenes (**16**–**22**).

Eleutherobin (**16**) is unique nitrogen-containing diterpene glycoside, isolated from an Australian soft coral, identified as *Eleutherobia* sp. (Figure 2). The non-sugar moiety of eleutherobin was escalated by presence of eunicellane nucleus, a diterpene framework that is first reported from the gorgonian octocoral *Eunicella stricta* [34]. It possesses *N*-methylurocanic acid ester at the C-8 position, while the sugar moiety is acetyl-arabinose [26,34,35]. Eleutherobin possessed significant cytotoxic effect towards various cancer cells with IC_50_ range of 10–15 nM. National Cancer Institute measured the selectivity of Eleutherobin (**16**) on 60 diverse panels of cancer cells. Its potency was estimated as 100-fold more against selected types of cancer cell lines than paclitaxel [36,37,38,39]. Investigation of a Caribbean octocoral, namely *Erythropodium caribaeorum*, collected from shallow reefs near Dominica, led to isolation of seven compounds: eleutherobin (**16**), desmethyleleutherobin (**17**), desacetyleleutherobin (**18**), isoeleutherobin A (**19**), *Z*-eleutherobin (**20**), caribaeoside (**21**), and caribaeolin (**22**) (Figure 2) [36]. The reported pharmacological mechanism of eleutherobin is mimetic to the mode of action observed with paclitaxel [37,38,39]. This appeared through stabilization of microtubules and confirmed by employing the electron microscopy technique [37,38,39]. 

This class of compounds (nitrogen-containing diterpenes, **16**–**22**), was examined as pharmacophore models for microtubule stabilizing agents. Desacetyleleutherobin (**18**) possesses the arabinose, instead of 2′acetyl substituent, while isoeleutherobin A (**19**) has an acetyl group at the 3′ instead of the 2′. *Z*-eleutherobin (**20**) is a geometric isomer of eleutherobin at the C-2′ to C-3′ double bond of the C-8 *N*-(6′)-methylurocanic acid ester side chain. Desmethyleleutherobin (**17**) differs from eleutherobin (**16**) by the occurrence of a hydroxyl function instead of an OMe at C-4. Caribaeoside (**21**) differs from eleutherobin by the presence of a hydroxyl at C-11 of the tricyclic core, and a double bond at C-12 to C-13 instead of C-11 to C-12, significantly altering the cyclohexene ring. Caribaeolin (**22**) differs from caribaeoside only by the occurrence of a –CH_2_OCO-CH_3_ substituent in the C-3 terminal chain. Eleutherobin (**16**) had an IC_50_ of 100 nM. The activity of *Z*-eleutherobin (**20**) was close, with an IC_50_ of 250 nM. Desmethyleleutherobin (**17**) and isoeleutherobin A (**19**) were more potent than eleutherobin, with IC_50_ of 20 and 50 nM, respectively, while Desacetyleleutherobin (**18**) was less potent, with an IC_50_ of 400 nM [35,36,37,38,39]. 

Sarcodictyins are congeners of eleutherobin and belong to a family of marine-derived diterpenoids with potent antitumor effects. They possess a rigid oxygen-bridged bi­ cyclo[8.4.0]tetradecatriene skeleton [40]. Sarcodictyins A and B (**23**–**24**) were isolated from stoloniferan coral *Sarcodictyon roseum* [41]. A year later, sarcodictyin C–F (**25**–**28**) were isolated from the same soft coral [42]. These metabolites showed their cytotoxic effects through tubulin binding in a mechanism similar to Taxol. Sarcodictyins A (**23**), B (**24**), C (**25**) and E (**27**) were proven to be more potent cytotoxic than the other sarcodictyins. Pharmacia-Upjohn′s researcher (1997) reported that sarcodictyins A–C (**23**–**25**) and E (**27**) exhibited cytotoxicity against Ll 210 murine leukemia (IC_50_ ranged between 408.5 ± 21.3 and 911.7 ± 393.5 nM; compare with taxol: IC_50_ = 16.6 ± 5.2 nM). The dose required to promote 90% tubulin polymerization ranged 1.7–6.6 µM, while 4.4 µM for taxol [40]. Five non-nitrogenous sarcodyctins have been isolated from *Alcyonium valdivae*: valdivone A (**29**), valdivone B (**30**), 4-*O*-Methyl valdivone A (**31**) 4-*O*-Methyl valdivone B (**32**), and dihydrovaldivone A (**33**) [43]. Compounds **29**–**30** were shown to inhibit the chemical induced anti-inflammatory. Sarcodictyin A (**23**) showed lower activity, with an IC_50_ of 2 μM, while caribaeoside (**21**) and caribaeolin (**22**) were considerably less potent, with an IC_50_ of 20 µM for both compounds [35,36,37,38,39]. 

Eleuthosides A (**34**) and B (**35**), along with sarcodictyin A (**23**) were reported from *Eleutherobia aurea*, gathered around Kwazulu-Natal Coast, South Africa [44]. Both **34** and **35** are novel diterpenoidal glycosides, and were proven to have potent microtubule stabilizing effect. The detailed information is registered in a patent with publication Number WO1999021862 A1.

**Figure 3:**
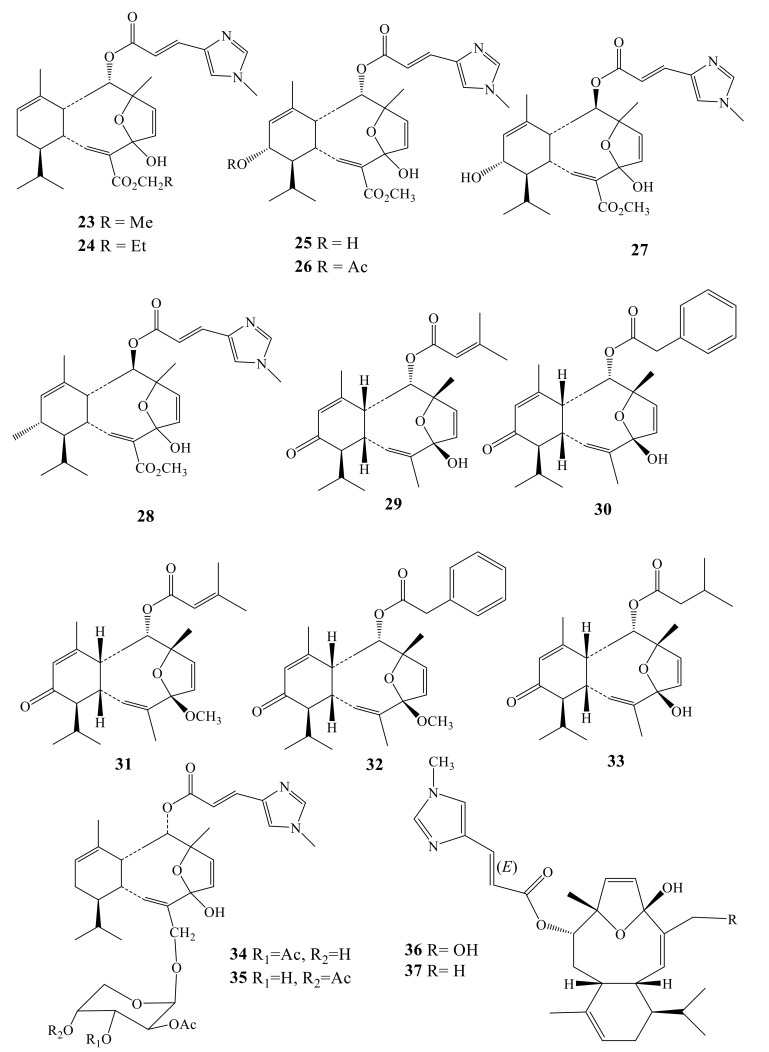
N-Containing diterpenes (**23**–**37**).

Investigation of another *Erythropodium* species, *E. caribaeorum*, led to identification of two unprecedented natural metabolites, namely caribaeorane (**36**) and 15-hydroxycaribaeorane (**37**), which are characterized by the presence of C-4 methylketal (Figure 3) [36].

**Figure 4:**
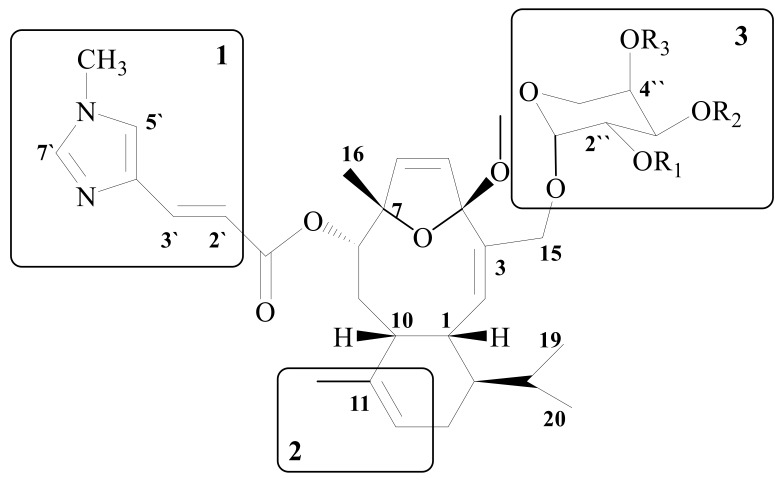
The three possible domains for binding to tubulin.

Eleutherobin has potent inducing in vitro tubulin polymerization effect, although its cytotoxicity is less than those obtained from paclitaxel [35,36,37,38,39,44]. P-glycoprotein is a target substance of Eleutherobin, similar to paclitaxel. Both showed cross-resistance in MDR1-expressing lines. Sarcodictyins are reported from *Sarcodictyon roseum* and seemed to be more promising than eleutherobin, despite their lower effects. Eleutherobins and sarcodictyins have been extensively modified by employing conventional and combinatorial chemistry techniques, which have also allowed the formation of hybrid molecules of the two base structures. These investigations indicated their SARS (Figure 4) [36,44]. 

The side chain and the imidazole ring are important. 

Both OH and OCH_3_ groups are tolerated, with little difference in effects. 

Elimination or alteration of the aglycone moiety of eleutherobin changes the cytotoxicity and resistance pattern. 

Sarcodictyin esters are more potent than amides.

**Figure 5:**
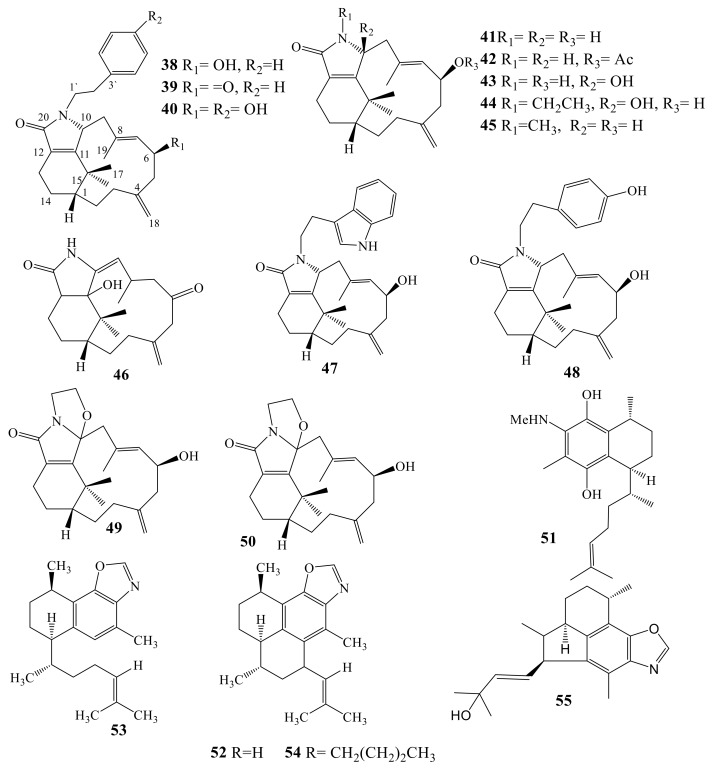
N-Containing diterpenes (**38**–**55**).

Cespitulactams A–C (**38**–**40**, Figure 5) are reported from *Cespitularia taeniata*, collected around Taiwan [45]. The obtained metabolites are nitrogenous tricyclic derivative, close to taxane diterpenes, particularly 3, 8-*seco*-taxoids and an infrequent *N*-phenylethyl-butyrolactamyl moiety. Four cancer cell lines, WiDr and Daoy, KB and Hepa59T/VGH, were tested. Cespitulactam A (**38**) exhibited potent toxicity against Widr and Daoy cancer cells (IC_50_ = 2.72 and 6.34 μg/mL, respectively) [46]. Further investigation of the organic extract of *Cespitularia taeniata* led to identification of eight more nitrogenous verticillene diterpenoids, cespitulactams (D–K) (**41**–**48**) [47]. These substances were evaluated against KB and murine L1210 leukemia. Compound **48** was active towards both cancer cells at 8.49 and 11.7 µM, respectively, and no effect was observed with the rest. Diterpene alkaloids (**41**–**48**) have been assayed for antimicrobial effects towards several microorganisms. Cespitulactam G (**44**) showed potent effect towards *Trichophyton mentagrophytes* (IFM45110) with MIC value of 11.56 µM. Compounds **41**, **47**, and **48** displayed significant effect towards *Micrococcus luteus* (IFM2066) and *Cryptococcus neoformans* (IFM46914) (**46**, **47** and **48**) with MIC values of 12.53, 9.06 and 9.54 µM, respectively, and *T. mentagrophytes* (**42** and **47**) with MIC values of 11.62 and 9.06 µM. Unfortunately, all isolated Cespitulactams (**41**–**48**) were inactive against *Staphylococcus aureus* (209P) [46]. Another investigation on *Cespitularia taeniata* led to identification of two nitrogenous verticillene (Figure 5), cespilamides A and B (**49** and **50**). Neither compound exhibited cytotoxicity against Hela, Daoy, WiDr, or MCF-7 at concentration >40 μM [34,47].

Diterpenoidal alkaloid, elisabethamine (**51**), has a serrulatane nucleus with methyl amino functionality, isolated from *Pseudopterogorgia elisabethae* [48]. Elisabethamine (**51**) had significant effect against LNCap and Calu cancer cell lines (IC_50_ = 10.35 and 20 μg/mL, respectively) [48]. Further investigation of the organic extract of *Pseudopterogorgia elisabethae* afforded pseudopteroxazole (**52**) and *seco*-pseudopteroxazole (**53**), having amphilectane skeleton and the uncommon benzoxazole function [49]. One more amphilectane derivative homopseudopteroxazole (**54**) was isolated from *Pseudopetrospongia elisabethae* collected from the Western Atlantic Ocean [50]. Pseudopteroxazole (**52**) showed potent inhibitory effect (97%) against *Mycobacterium tuberculosis* H37Rv at 40.45 µM, whereas *seco*-pseudopteroxazole (**53**) inhibited 66% of mycobacterial growth. Therefore, the potency effect of **52** might be attributed to the benzoxazole function. Remarkably, biological screening of **52** against 60 cancer cell lines designated potent in vitro cytotoxicity. Several approaches for syntheses of pseudopteroxazole are published [51,52,53,54]. Another diterpene, ileabethoxazole (**55**), was obtained as a light yellow oil from *Pseudopterogorgia elisabethae* collected near Providencia Island (Figure 5). It represents novel diterpenes carbon skeleton (Ileabethane), which appears to be biosynthetically related to the serrulatane skeleton. It displayed strong inhibitory effect against *M. tuberculosis* H37Rv [55,56,57].

Three new nitrogen containing cembranolide diterpenes, sinularamine I (**56**), sinularamine II (**57**), and sinularamine III (**58**), each possessing a dimethylamino group, were isolated from the Okinawan soft coral species of *Sinularia* genus and their structures were elucidated by spectroscopic analyses and chemical transformations [58]. Compounds **56** and **58** inhibited the proliferation of KB cells at concentrations of 2.0 and 1.65 µg/mL, respectively [58,59]. Modified structures of sinularamines I–III were isolated from Okinawan soft coral of *Sinularia* species [59,60].

Investigation on *Lobophytum* sp., collected from Philippine Islands, led to identification of a cembranoid diterpene (17-dimethylaminolobohedleolide, **59**), which blocked the cytopathic effect of in vitro HIV-1 infection in a cell-based assay with EC_50_ = 8.78 µM [61].

Tinto et al. (1991) reported seven pseudopteranoids isolated from *Pseudopterogorgia acerosa*, including the rare bisditerpenoid amine bis(gorgiacerol)amine (**60**). It showed selective growth inhibition activity against cancer cell lines (HCT116 and HeLa) [62,63].

Caucanolide B (**61**), a possible pesudopterane-type diterpenoid successor through oxidation cleavage at C-2/C-3 together with five rare diterpenes, was isolated from *Pseudoptergorgia bipinnata* collected near the Colombian Southwestern Caribbean Sea. These caucanolides were evaluated against the malaria parasite, *Plasmodium falciparum*. Unfortunately, Caucanolide B showed no significant activity, although it is the only example from nature of a secondary metabolite possessing the *N*1,*N*1-dimethyl-*N*2- acylformamidine functionality [64].

**Figure 6:**
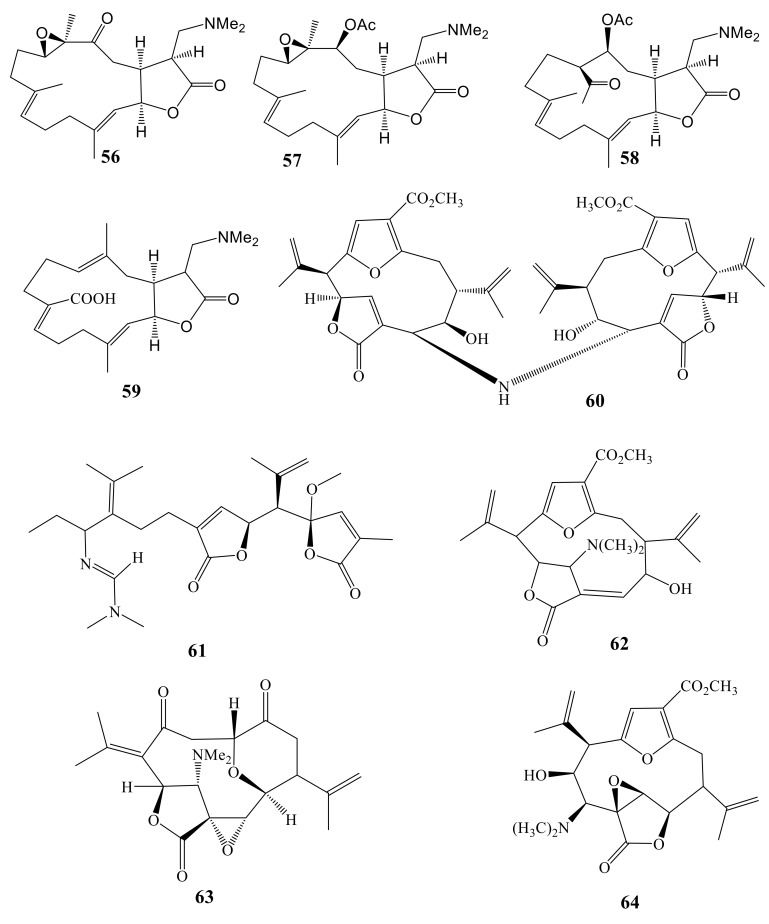
N-Containing diterpenes (**56**–**64**).

Fenical and Clardy (1982) examined the constituents of Floridian specimens of *Pseudopterogorgia acerosa*, leading to the isolation of pseudopterolide, a remarkable metabolite based on the 12-membered carbocyclic pseudopterane skeleton [64]. Further examination of extracts of several *Pseudopterogorgia* spp. has since resulted in the isolation of other pseudopterane metabolites, many of which possess chemically unique structural features (Figure 6). Tobagolide (**62**), one such metabolite, is a rare nitrogen-containing diterpenoid isolated from a Trinidadian specimen of *Pseudoptergorgia acerosa* [65]. Another examination of Puerto Rican specimens of *Pseudoptergorgia acerosa* led to isolation and structural determination of alanolide (**63**), a novel tetracyclic norditerpene, which appears to be biogenetically related to tobagolide [66]. The structure of aceropterine (**64**), the first pseudopterane with a transposed lactone moiety, is closely related to that of tobagolide, which was isolated from a Trinidadian specimen of *Pseudoptergorgia acerosa* [67,68].

### 2.3. Terpenoidal Alkaloids

Zoanthamine-type alkaloid (Lobozoantamine, **65**) was identified from an Indonesian coral, *Lobophytum* sp. It belongs to a unique class of alkaloids, the precursor type of which is still ambiguous, whether it is a triterpenoid or a polyketide [68]. The first member of the zoanthamine-type alkaloid was isolated from *Zoanthus* sp. [69,70], followed by a series of analogs isolated from the genus *Zoanthus*, with the single exception of zooxanthellamine, which was isolated from the unicellular dinoflagellate *Symbiodinium* sp. [71]. Lobozoanthamine (**65**) was evaluated against AGS and C6, and showed cytotoxic effect with IC_50_ value > 50 μM on both cell lines. 

**Figure 7:**
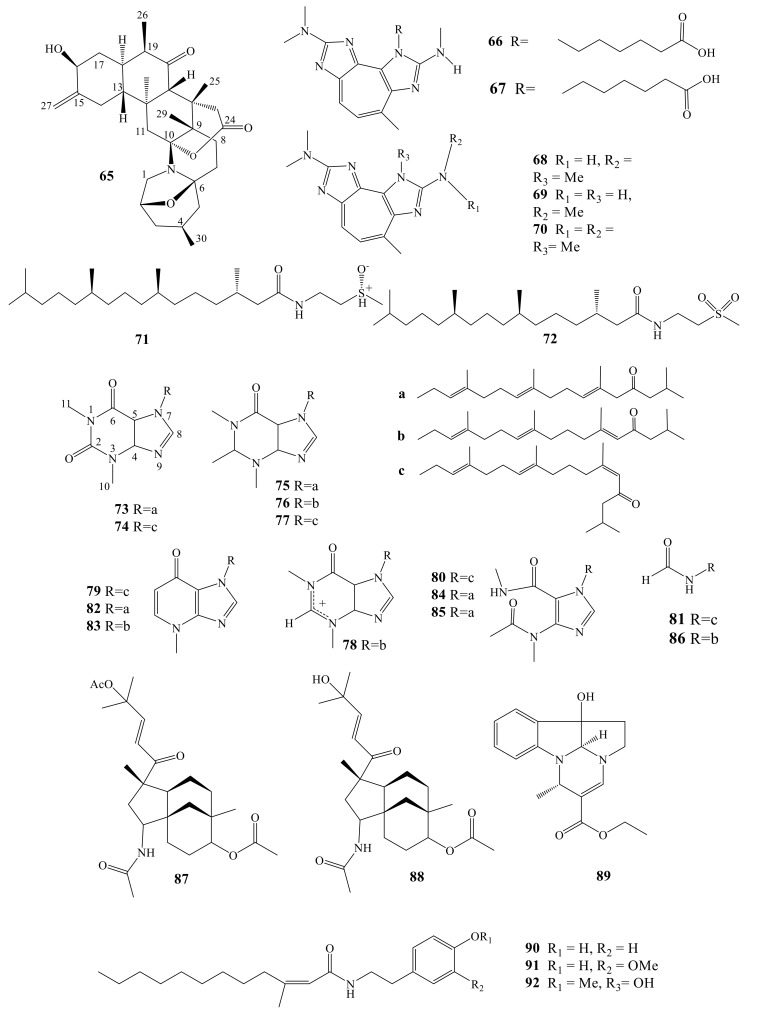
Terpenoidal alkaloids (**65**–**92**).

Five zoanthoxanthin alkaloids (Figure 7) were reported from Chinese Sea gorgonian *Echinogorgia pseudossapo*, pseudozoanthoxanthins III (**66**), pseudozoanthoxanthins IV (**67**), Zoanthoxanthin (**68**), Paragracine (**69**) and Zoanthoxanthin (**70**) [72]. In vitro antiviral activity of **66**–**69** against HSV-1 was evaluated using plaque reduction assay. The completely non-toxic concentration of **66**–**69** and positive control ACV on Vero cells were tested to be 270.3, 523.6, 185.2, 195.3, and >7500 μM by MTT assay, respectively. Further antiviral studies, employing concentrations lower than the tested non-toxic concentration values. Compounds **66**–**69** exhibited anti-HSV-1 activity with EC_50_ values of 108.1, 471.2, 70.4, 117.2, and 6.08 μM, respectively. The results suggest that the side chain at the nitrogen *N*(3) in **66**–**69** could affect their antiviral activity. Although **66** and **68** showed mild anti-HSV-1 activity, their effects were less than that obtained with the positive control [72].

Sinulasulfoxide (**71**) and sinulasulfone (**72**), two alkaloids with sulfur moiety, were reported from Indonesian *Sinularia* sp. [73]. Their skeleton is characterized by presence of a phytanic acid moiety connected to sulfated diterpene through an amide bond. Among marine metabolites, the 2-methylsulfinyl ethanamine moiety infrequently occurs in nature; however, it was isolated by Piper [74]. Another example, psammaplin N, was isolated from sponge *Aplysinella rhax* [72]. An analog, 3-methylsulfinyl propanamine (decarboxylated methionine sulfoxide), was also reported from the same sponge [74]. Both compounds were evaluated for anti-inflammatory activity. Sinulasulfoxide (**71**) showed moderate inhibition of NO_2_ production (22.6%) [72].

Nuttingins A–F (tetraprenylated purine alkaloids, **73**–**78**) and malonganenones D–H (**82**–**86**) were reported from *Euplexaura nuttingi* [75], while malonganenones, A–C (**79**–**81**) were previously reported from *Leptogorgia gilchristi* [76]. Nuttingins A–E (**73**–**77**) and malonganenones D–G (**82**–**84**) showed inhibitory effect towards K562 and UT7 cancer cells (Figure 6). 

Compounds **73**–**77** and **82**–**86** also induced apoptosis in transformed mammalian cells at 1.25 µg/mL [5,76]. Nuttingin A (**73**) is the first 3, 7-disubstituted hypoxanthine, while Nuttingin B (**74**) and Nuttingin C (**75**) represent the first formamides identified from soft corals. The anti-esophageal cancer effect of Nuttingins A–C (**73**–**75**) was compared with that of rietone, which was previously identified from *L. gilchristi*. Nuttingins A–C (**73**–**75**) were assessed towards several tumor cells (WHCO1, WHCO5, WHCO6, KYSE70, KYSE180, and KYSE520). Compounds **73**–**75** showed moderate cytotoxicity against seven cancer cells. Remarkably, Nuttingin A (**73**) exhibited toxicity against WHCO1 cells comparable with cisplatin (IC_50_ =15 μM). The anti-microbial effect of Nuttingins A (**73**) was evaluated against *Staphylococcus aureus*, *Escherichia coli*, and *Aspergillus niger* (20 and 100 mg/disk). Nuttingins A–E (**73**–**77**) showed no effect against *E. coli* and *A. niger*, while showed mild effect against *S. aureus* (100 mg/disk) [5,77].

Two unprecedented nitrogenous diterpenoidal metabolites, sarinfacetamides A (**87**) and B (**88**), were reported from the Chinese *Sarcophyton infundibuliforme* [78]. These compounds are members of uncommon tricycle-dodecane scaffold with an acetamide group (Figure 6). Compounds **87** and **88** were evaluated for different biological effects, including cytotoxicity and immunomodulatory effects. Unfortunately, when evaluated against HL-60, K562, MGC-803, BEL-7402, SH-SY5Y, HCT-116, MDAMB-231, A549, MCF-7/ADM, HO8910, U87, and NCIH1975 cell lines, they showed no effects at 10 μM. Interestingly, in the immunological assay, compound **87** was found to moderately promote the ConA-induced T lymphocytes at 10 μM with proliferation rate of 36.18% [78]. 

A rare pyrroloindoline alkaloid verrupyrroloindoline (**89**) was isolated from *Sinularia verruca*. It showed no protection towards the cytopathic effects of HIV-1 infection and no effect was observed against LPS-induced NO production [79].

4-(2-aminoethy1)-2-methoxyphenol (**90**), 5-(2-aminoethy1)-2-methoxyphenol (**91**) and (2)-3-methyldodec-2-enoic acid (**92**), three tyramine derivatives, were isolated from the soft coral *Sinularia flexibilis* (Figure 7). It is worth mentioning that total synthesis was the main tool for structural elucidation of these amides [80]. Compound **90** displayed potent anti-inflammatory effect in the inhibition of superoxide generation and elastase release in fMLP/CB-induced human neutrophils [80]. 

### 2.4. Ceramides and Cerebrosides

Cerebrosides are a group of metabolites holding a ceramide moiety and sugar units [81]. They have been published from different invertebrates including, sponges [82], sea anemones, [83], ascidians [84] and soft corals [85,86,87,88,89,90,91,92,93,94]. 

**Figure 8:**
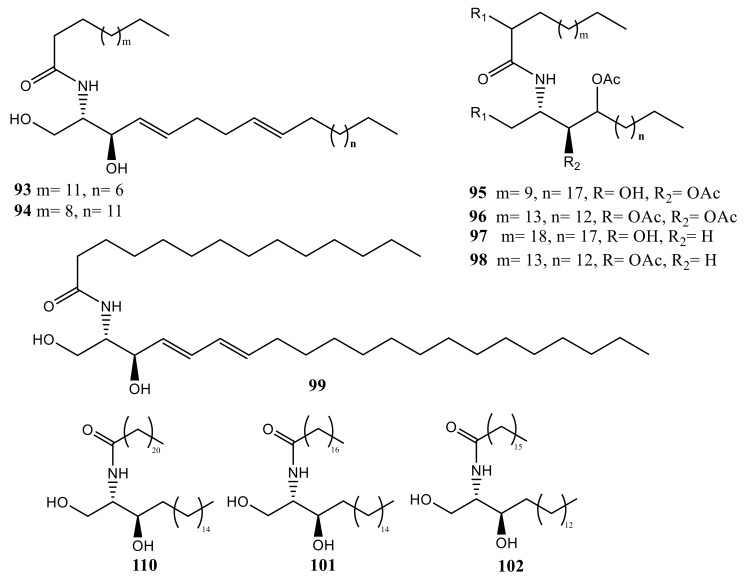
Sphingolipids (**93**–**102**).

Investigation of *Sinularia crassa* led to identification of two new sphingosine derivatives, *N*-hexadecanoyl-1,3-dihydroxy-2-amino-4,8-octadecadiene (**93**) and *N*-heneicosanoyl-1,3,4-trihydroxy-2-aminotetradecane (**94**) [85]. Two sphingolipids, (2*S*,3*S*,4*R*)-1,3,4-trihydroxy-2-[((*R*)-2′-hydroxytetradecanoyl) amino] tricosane (**95**) and (2*S*,3*S*,4*R*)-1,3,4-triacetoxy-2-l((*R*)-2′–acetoxy octadecanoyl) amino] octadecane (**96**), were isolated from *Sinularia leptoclados* (Figure 8). They exhibited mild antibacterial effect against Gram-negative bacteria, while no activity was found against Gram-positive bacteria tested [86]. 

Investigations of *Lobophytum* sp. yielded a ceramide: (2*S*,3*S*,4*R*)-2-[(*R*)-2′-hy-droxytricosanoyl amino]-1,3,4-tridecanetriol (**97**). The length of the sphingosine chain of the fatty acid was determined by methanolysis, followed by acetylation of sphingamines to give **98** [87].

Investigation of *Sarcophyton auritum* led to identification ceramide *N*-((2*S,*3*R*,4*E*,6*E*)-1,3-dihydroxyhenicosa-4,6-dien-2-yl) tridecanamide (**99**), which showed antagonized effect the lethality of pentylenetetrazole in mice. In addition to this effect, compound **99** showed significant anxiolytic effect as well as CNS depressing activity, possibly through GABA and serotonin receptor modulation [88]. 

Three sphingosines, (*2S,3R*)-2-(docosanoyl amino)nonadecane-1,3-diol (**100**) and (2*S*,3*S*,4*R*)-2-[(29*R*)-29-hydroxynonadecanoylamino]nonadecane-1,3,4-triol (**101**), along with the known (2*S*,3*R*,4*E*)-2- (heptadecanoylamino)octadec-4-ene-1,3-diol (**102**), were isolated from *Pseudopterogorgia australiensis* collected from the Tuticorin Coast, India (Figure 7) [89]. Compounds **100**–**102** showed moderate antibacterial activity against Gram-positive bacteria *Bacillus pumilis*, *B. subtilis*, and *S. aureus* and Gram-negative bacteria *E. coli*, *Proteus vulgaris*, and *Pseudomonas aeruginosa*. Unfortunately, no significant effect was observed towards *Candida albicans* or *Aspergillus niger* [89].

Investigation of *Sarcophyton ehrenbergi* collected from water around Taiwan led to cerebrosides sarcoehrenoside A (**103**), sarcoehrenoside B (**104**), and known ceramide (**105**), along with three known cerebrosides (**106**–**108**) [90]. Sarcoehrenoside A (**103**) has a characteristic β-glucose moiety, which differs from previous reported marine cerebrosides (Figure 8). Compounds **103**–**108** were evaluated toward their antimicrobial effects including *Enterobacter aerogenes, Serratia marcescens*, *Salmonella enteritidis, Yersinia enterocolitica* and *Shigella sonnei*. Unfortunately, they showed no antimicrobial effect (100 μg/disk). However, in-vitro anti-inflammatory effects of **103**–**108** were estimated [90]. Both **103** and **108** reduced the levels of iNOS to 46.9 ± 9.7% and 20.3 ± 6.8%, respectively, and of COX-2 to 77.2 ± 9.9% and 64.3 ±8.6%, respectively. Compounds **104, 105,** and**10**7 reduced iNOS protein expression to be ranged 25.8- 47.3%, did not inhibit COX-2 protein expression [90].

**Figure 9:**
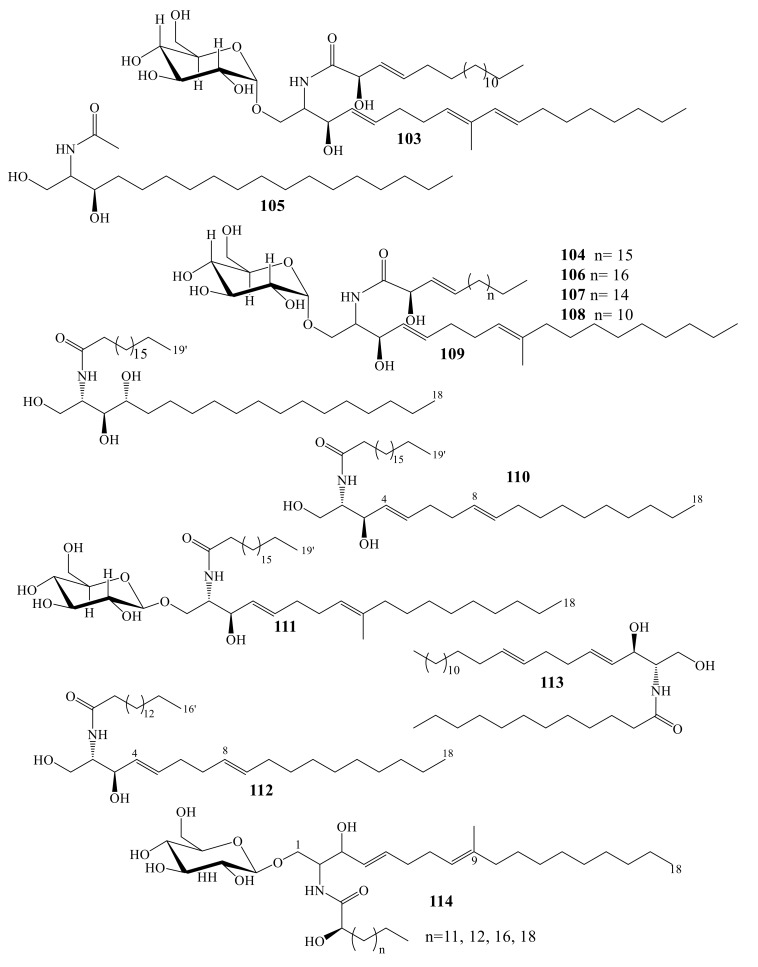
Ceramides and Cerebrosides (**103**–**114**).

Four sphingolipids were isolated from *Lobophytum* sp. (Figure 9), identified as (2*S*,3*S*,4*R*)-2- nonadecanoylamino-octadecane-1,3,4-triol (**109**), (2*S*,3*R*,4*E*,8*E*)-[(2′*R*)-2′-hydroxyhepta decanoylamino]-4,8-octadecadiene-1,3-diol (**110**), 1-*O*-(β-d-glucopyranosyl)-(2*S*,3*R*,4*E*,8*E*)-2-[(*2*′*R*)-2′-hydroxynonadecanoyl amino]-9-methyl-4,8-octadecadiene-1,3-diol (**111**), and (2*S*,3*R*,4*E*,8*E*)-2-hexadecanoyl amino-4,8-octadecadien-1,3-diol (**112**). All these sphingolipids were evaluated for toxicity against human Peripheral Blood Mononuclear Cells (PBMC) [91]. 

Bioactivity directed purification of the *Litophyton arboreum* extract led to the identification of erythro-*N*-dodecanoyl-docosasphinga-(4*E*,8*E*)-dienine (**113**) and showed inhibitory effect against HIV-1 PR (IC_50_ = 4.80 ± 0.92 μM). Compound **113** showed potent HIV-1 PR inhibitory effect in the absence of cytotoxicity against the tested cell lines [92]. Compound **113**, which was previously isolated from *Anemonia sulcata*, showed cytotoxicity (ED_50_ of 37.31 μM [83].

Anti-inflammatory cerebroside (**114**) was isolated from Indonesian *Sinularia* sp. [93]. The ESI-MS spectrum of **114** indicated it to be a mixture of homologs (Figure 8). Micro-scale chemical degradation of **114** allowed indicating the nature of the fatty acid moieties present [93,94]

### 2.5. Miscellaneous Nitrogen-Containing Metabolites

Spermidine is a polyamine found in ribosomes and living tissues. It has various metabolic functions within organisms and was isolated originally from semen. Spermidine is commonly used for in vitro molecular biology reactions, particularly in vitro transcription by Phage RNA polymerases, in vitro transcription by human RNA polymerase II, and in vitro translation. Spermidine increases specificity and reproducibility of Taq-mediated PCR by neutralizing and stabilizing the negative charge on DNA phosphate backbone. Spermidine is, at physiological pH, a polycationic reagent that aids in enzyme digestion by forcing apart DNA molecules. Two cytotoxic spermidine derivatives (**115** and **116**) (Figure 9) were reported from Pacific *Sinularia brongersmai* [95]. Sinulamide (**117**), a tetraprenylatedspermine derivative, was identified from Japanese *Sinularia* sp. Sinulamide (**117**) not only inhibits H, K-ATPase with an IC_50_ value of 5.5 µM, but also is cytotoxic against L1210 and P388 with IC_50_ values of 3.1 and 4.5 µg/mL, respectively [96].

Two *N*-methylated spermidine amides, **118** and **119**, were isolated from *Sinularia* sp. [97]. An acylated spermidine (**120**) was identified from *Sinularia* sp. and was found to be cytotoxic against P-388 cells (ED50 0.04 µg/mL) [98]. 

A pyrazine congener, clavulazine (**121**), was first isolated from isolated from Okanawan *Clavularia viridis* [99], along with two marine pyrazine derivatives, Clavulazols A (**122**) and B (**123**) [100]. 

**Figure 10:**
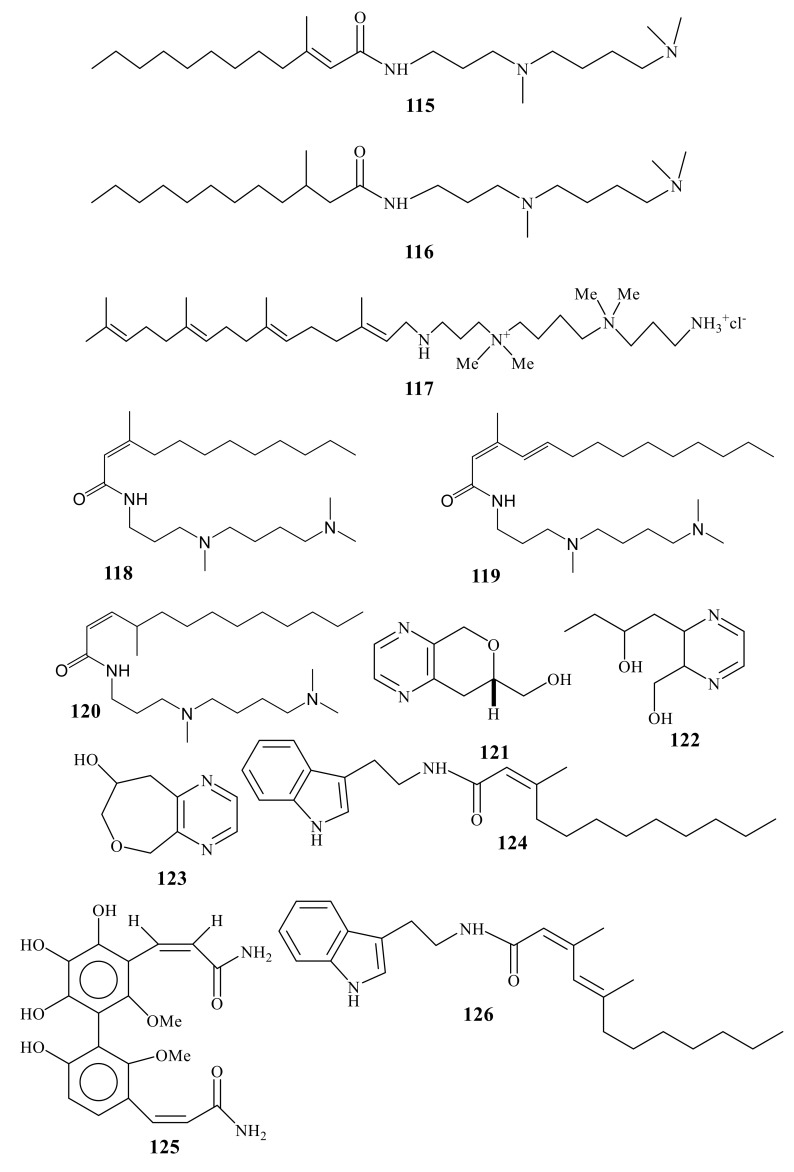
Spermidines (115–126).

A unique cinnamide dimer (**124**) was isolated from *Sinularia flexibilis* [101]. Granulatamides A (**125**) and B (**126**) and a tryptamine derivative (Figure 10) were isolated from the *Eunicella granulate* (Figure 10). The cytotoxic effects of **125** and **126** were evaluated against certain cancer cell and GI_50_ values were in ranges 1.7–12.7 and 3.5–13.8 µM, respectively [102].

## 3. Summary and Conclusions

Natural products possess a characteristic chemical spatial orientation. This enables them to interact with their biological targets, which validates initial points for drug discovery. Recently, half of new drugs reported are naturally occurring or constructed on the basis of natural chemical frame. Forty percent of the bioactive compounds are natural metabolites and appear in the Dictionary of Natural Products. Chemical novelty of marine products is superior to terrestrial metabolites. Approximately 70% of the molecular skeletons that appear in databases are produced by marine organisms. Additionally, marine drugs have successfully been purchased and others are in different clinical phases. 

**Figure 11:**
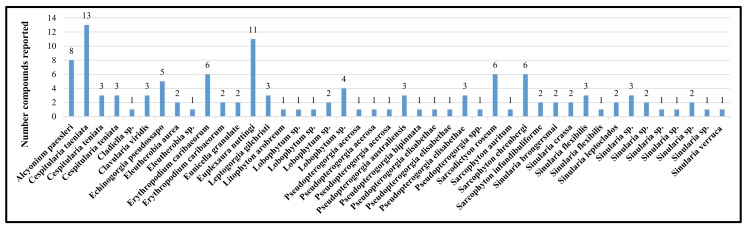
Distribution of Nitrogen containing metabolites between Alcyonacea species.

**Figure 12:**
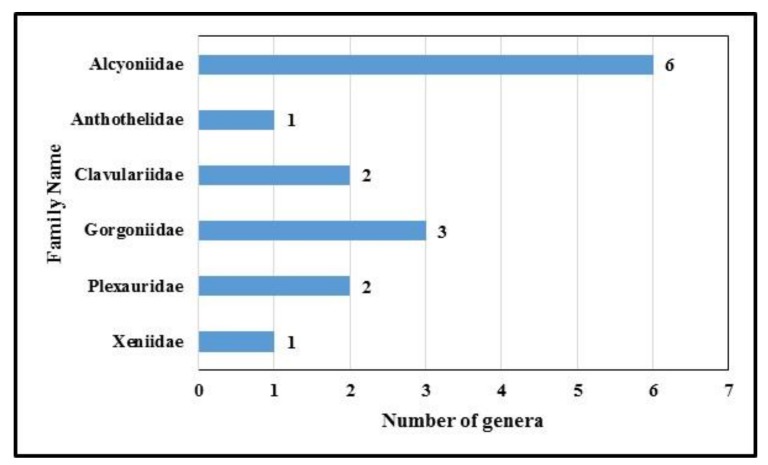
Relationship between Alcyonacea families and genera produce Nitrogen containing metabolites.

Alcyonacea will be considered as potential source of bioactive nitrogen containing metabolites. The engagement of different approaches played a significant role in the facilitation of the forthcoming drug discovery process. Noteworthy, many marine metabolites displaying fascinating molecular structures with diverse pharmacological effects have been reported from Alcyonacea during the last four decades (1978–2018). Of the 121 distinctive structures accounted for in this review, 61 (50.4%) are nitrogen-containing terpenoidal metabolites. Figure 11 illustrates the number of nitrogenous metabolites, as reported from 44 species. These species belong to six genera, as illustrated in Figure 12.

**Figure 13:**
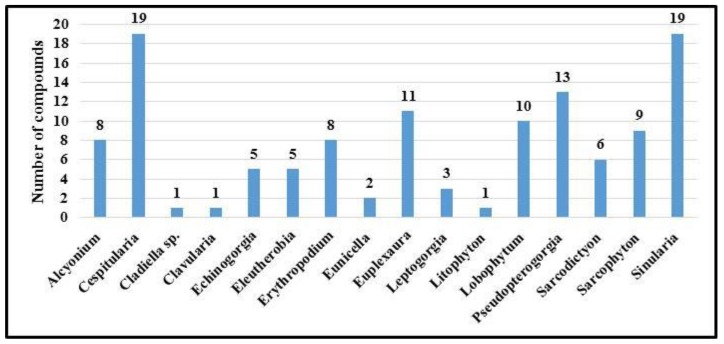
Distribution of nitrogen containing metabolites isolated from soft corals by genus.

Figure 13 illustrates nitrogenous metabolites produced by 15 genera. The five most productive genera are: *Cespitularia, Expeuxaura, Lobophytum*, *Pseudopterogorgonia* and *Sinularia*, with 19 (15.7%), 11 (9%), and 10 (8%), 13 (9.9%) and 21 (17.3%), respectively.

A remarkable 121 metabolites reported from Alcyonacea are discussed in the current review. Of these distinctive structures, 121 (100%) are nitrogen-containing terpenes. The major classes of nitrogen-containing metabolites produced by Alcyonacea are sesquiterpenes, diterpenes and ceramides. The estimated analysis per class is as follows: 46 (38%) are diterpenes, 15 (12.4%) are sesquiterpenes, 24 (19.8%) are alkaloids and the remaining 36 (28.8%) are spermidins, cerebrosides, and ceramides, as appeared in Table 2.

Diterpenoids, the major division, are in turn further analyzed for each Alcyonacean species, and the distribution by skeleton classes of compounds in this group is shown in Table 3, which summarizes the impressive structural variety of terpenoid carbon skeletons found in these animals. The diterpene skeleton is most frequently elaborated by the genera *Pseudopterogorgia, Erythropodium, Sarcodictyon, Lobophytom* and *Sinularia.* These diterpenes have highly functionalized moieties with potent cytotoxicity that could be mimetic the mode of action of taxol. Some of them are currently in clinical trials. 

## Figures and Tables

**Table 1:** Bioactivity of nitrogen containing metabolites from Alcyonacea.

Cpd. No.	Species	Bioactivity	Ref. No.
**1–8**	*Alcyonium paessleri*	Cytotoxic	[20]
**9**	*Cladiella* sp.	Not published data	[21]
**10–12**	*Cespitularia taeniata*	Cytotoxic	[31,32]
**13–15**	*Cespitularia taeniata*	Cytotoxic	[33]
**16**	*Eleutherobia* sp.	Cytotoxic	[34]
**17–22**	*Erythropodium caribaeorum*	Cytotoxic	[35,36,37,38,39]
**23–28**	*Sarcodictyon roseum*	Cytotoxic	[40,41,42]
**29–33**	*Alcyonium valdivae*	Anti-inflammatory	[43]
**34–35**	*Eleutherobia aurea*	Cytotoxic	[44]
**36–37**	*Erythropodium caribaeorum*	Cytotoxic	[36]
**38–50**	*Cespitularia taeniata*	Cytotoxic and Antimicrobial	[45,46,47]
**51**	*Pseudopterogorgia elisabethae*	Cytotoxic	[48]
**52–54**	*Pseudopterogorgia elisabethae*	Cytotoxic	[49,50]
**55**	*Pseudopterogorgia elisabethae*	Cytotoxic	[51,52,53,54,55,56,57]
**56–58**	*Sinularia* sp.	Antiproliferative	[58,59]
**59**	*Lobophytum* sp.	Cytotoxic	[60,61]
		HIV inhibitory	
**60**	*Pseudopterogorgia acerosa*	Cytotoxic	[62,63]
**61**	*Pseudopterogorgia bipinnata*	Antimalarial	[64]
**62**	*Pseudopterogorgia acerosa*	-	[65]
**63**	*Pseudopterogorgia acerosa*	-	[66]
**64**	*Pseudopterogorgia* spp.	Cytotoxic	[67,68]
**65**	*Lobophytum* sp.	Cytotoxic	[69,70,71]
**66–70**	*Echinogorgia pseudossapo*	Antiviral	[72]
**71–72**	*Sinularia* sp.	Inhibit LPS-induced NO release	[73,74]
**73–86**	*Euplexaura nuttingi* and *Leptogorgia gilchristi*	Cytotoxic	[75,76,77]
**87–88**	*Sarcophyton infundibuliforme*	Immunomodulator	[78]
**89**	*Sinularia verruca*	-	[79]
**90–92**	*Sinularia flexibilis*	Cytotoxic	[80]
**93–94**	*Sinularia crassa*	-	[81,82,83,84,85]
**95–96**	*Sinularia leptoclados*	Antibacterial	[86]
**97–98**	*Lobophytum* sp.	-	[87]
**99**	*Sarcophyton auritum*	Anxiolytic effect	[88]
**100–102**	*Pseudopterogorgia australiensis*	Antimicrobial	[89]
**103–108**	*Sarcophyton ehrenbergi*	Anti-inflammatory	[90]
**109–112**	*Lobophytum* sp.	Cytotoxic	[91]
**113**	*Litophyton arboreum*	HIV-1 PR inhibitory	[92]
**114**	*Sinularia* sp.	Anti-inflammatory	[93,94]
**115–116**	*Sinularia brongersmai*	Cytotoxic	[95]
**117**	*Sinularia* sp.	Gastric H, K-ATPase and cytotoxic	[96]
**118–119**	*Sinularia* sp.	Cytotoxic	[97]
**120**	*Sinularia* sp.	Cytotoxic	[98]
**121–123**	*Clavularia viridis*	Cytotoxic	[99,100]
**124**	*Sinularia* flexibilis	Cytotoxic	[101]
**125–126**	*Eunicella granulate*	Cytotoxic	[102]

**Table 2:** Distribution of nitrogen-containing metabolites isolated from soft corals by skeletal class.

Organism	Compounds	Chemical Class
*Alcyonium paessleri*	**1–8**	Illudalane-sesquiterpenoid
*Cladiella* sp.	**9**	Oxazole-derived sesquiterpene
*Cespitularia taeniata*	**10–15**	Eudesmane-sesquiterpenoid
	**38–50**	Verticillane-diterpenoid
*Eleutherobia* sp. and *Eleutherobia aurea*	**16,34, 35**	Eunicellane-diterpenoid
*Erythropodium caribaeorum*	**17–22, 36, 37**	Eunicellane-diterpenoid
*Sarcodictyon roseum*	**23–28**	Eunicellane-diterpenoid
*Pseudopterogorgia elisabethae*	**51**	Serrulatane-diterpenoid
	**52–54**	Amphilectane-diterpenoid
	**55**	Ileabethane-diterpenoid
*Pseudopterogorgia acerosa*	**60**	Pseudopteranoids bisditerpenoids
*Pseudopterogorgia bipinnata*	**63–64, 61**	Pesudopterane-diterpenoid
*Pseudopterogorgia* sp.	**62**	Pesudopterane-diterpenoid
*Pseudopterogorgia australiensis*	**100–102**	Ceramides
*Sinularia* sp.	**56–58**	Cembranoid-diterpenoid
	**71–72**	Geranylgeraniane-diterpenoid
*Sinularia verruca*	**89**	Pyrroloindoline alkaloid
*Sinularia* crassa, *Sinularia leptoclados*, and *Sinularia* sp.	**93–94,114**	Ceramides and cerebrosides
*Sinularia brongersmai* and *Sinularia* sp.	**115–120**	Spermidines
*Sinularia flexibilis*	**90–92**	Tyramine derivatives
*Lobophytum* sp.	**59**	Cembranoid-diterpenoid
	**65**	Zoanthamine-type alkaloid
	**88–112**	Ceramides and cerebrosides
*Litophyton arboreum*	**113**	Ceramides
*Echinogorgia pseudossapo*	**66–70**	Zoanthoxanthin alkaloids
*Euplexaura nuttingi*	**73–78**	Hypoxanthine
*Leptogorgia gilchristi*	**79–86**	Hypoxanthine and *Seco*-Hypoxanthine
*Sarcophyton infundibuliforme*	**87–88**	Tricyclic dodeca-diterpenoid
*Sarcophyton auritum*	**99**	Ceramides
*Sarcophyton ehrenbergi*	**103–108**	Ceramides and Cerebrosides
*Clavularia viridis*	**124**	Spermidines
*Eunicella granulate*	**125–126**	Spermidines

**Table 3:** Distribution of nitrogen-containing metabolites isolated from soft corals by genus and chemical classes.

Genus	Family	Common N-Containing Chemical Class
*Alcyonium paessleri* *Cladiella* sp. *Eleutherobia* *Sinularia* *Lobophytum* *Sarcophyton*	Alcyoniidae	Illudalane-type sesquiterpenes Oxazole-derived sesquiterpene Eunicellane- diterpenoid Cembranoid-diterpenoid Geranylgeraniane-diterpenoid Pyrroloindoline alkaloid Zoanthamine-type alkaloid Tricyclic dodeca-diterpenoid Ceramides and cerebrosides Spermidines
*Pseudopterogorgia* *Leptogorgia* *Eunicella*	Gorgoniidae	Serrulatane- diterpenoid Amphilectane- diterpenoid Ileabethane- diterpenoid Pseudopteranoids-bisditerpenoids Hypoxanthine and *seco*- Hypoxanthine Spermidines
*Cespitularia*	Xeniidae	Eudesmane- sesquiterpenoid Verticillane- diterpenoid
*Erythropodium*	Anthothelidae	Eunicellane- diterpenoid
*Sarcodictyon* *Clavularia*	Clavulariidae	Eunicellane- diterpenoid Spermidines
*Euplexaura*	Plexauridae	Hypoxanthine
